# Decision making of iatrogenic coronary embolism after SAVR: a case report

**DOI:** 10.1186/s13019-024-02613-1

**Published:** 2024-03-15

**Authors:** Atsuyuki Mitsuishi, Kazumasa Orihashi, Yujiro Miura, Ren Saito

**Affiliations:** 1 Department of Cardiovascular Surgery, Kochi Medical School Hospital, 185-1, Kohasu, Nankoku-shi, Okohmachi, Kochi Prefecture 783-8505 Japan; 2 Department of Anesthesiology and Intensive Care Medicine, Kochi Medical School Hospital, 185-1, Kohasu, Nankoku-shi, Okohmachi, Kochi Prefecture 783-8505 Japan

**Keywords:** Acute coronary syndrome, Coronary artery obstruction, Surgical aortic valve replacement, Transesophageal echocardiography, Aortic stenosis, Case report

## Abstract

**Background:**

Acute coronary artery obstruction is a rare but lethal complication of surgical aortic valve replacement (SAVR), which may be caused by embolization of resected native tissue such as calcium plaque, thrombus, or perivalvular aortic tissue like fat embolus. Coronary artery bypass grafting (CABG) and percutaneous coronary intervention (PCI) are the main treatment modalities. PCI is less invasive, but it is difficult to determine its feasibility intraoperatively.

**Case presentation:**

We report an 86-year-old woman who had asymptomatic severe aortic stenosis. She had scleroderma with an intractable left leg ulcer and bilateral leg varices. Considering the possibility of the spread of infection from the leg wound, SAVR was performed via right anterior thoracotomy to avoid complications such as mediastinitis. Coronary artery occlusion was suspected after weaning of cardiopulmonary bypass in the operation room due to asynergy with ST elevation and new severe mitral regurgitation. Transoesophageal echocardiography (TEE) helped diagnose coronary obstruction by embolus based on the degree of stenosis and the movement of the stenosis site. Percutaneous catheter intervention was performed successfully to restore coronary perfusion.

**Conclusion:**

TEE facilitated the diagnosis of coronary artery stenosis caused by an embolus and helped in determining the feasibility of percutaneous catheter intervention, thus allowing us to choose PCI over CABG as a less invasive surgery. This is especially invaluable in cases where obtaining a saphenous graft for CABG is difficult or where CABG would have required conversion from minimally invasive surgery (anterolateral approach) to median sternotomy.

## Introduction

Acute coronary artery obstruction is a rare but lethal complication of surgical aortic valve replacement (SAVR) [1] caused by embolization of the resected native tissue such as calcium plaque, thrombus, or the perivalvular aortic tissue (fat embolus). We report a case of acute coronary artery obstruction post-SAVR caused by an embolus that was quickly diagnosed by transesophageal echocardiography (TEE). The use of TEE helped avoid coronary artery bypass grafting (CABG) in this patient who had an intractable left leg ulcer and bilateral varices. Although TEE could not visualize the embolus directly, we were able to detect anomalies and indirectly predict the properties of the embolus.

## Case presentation

**Fig. 1:**
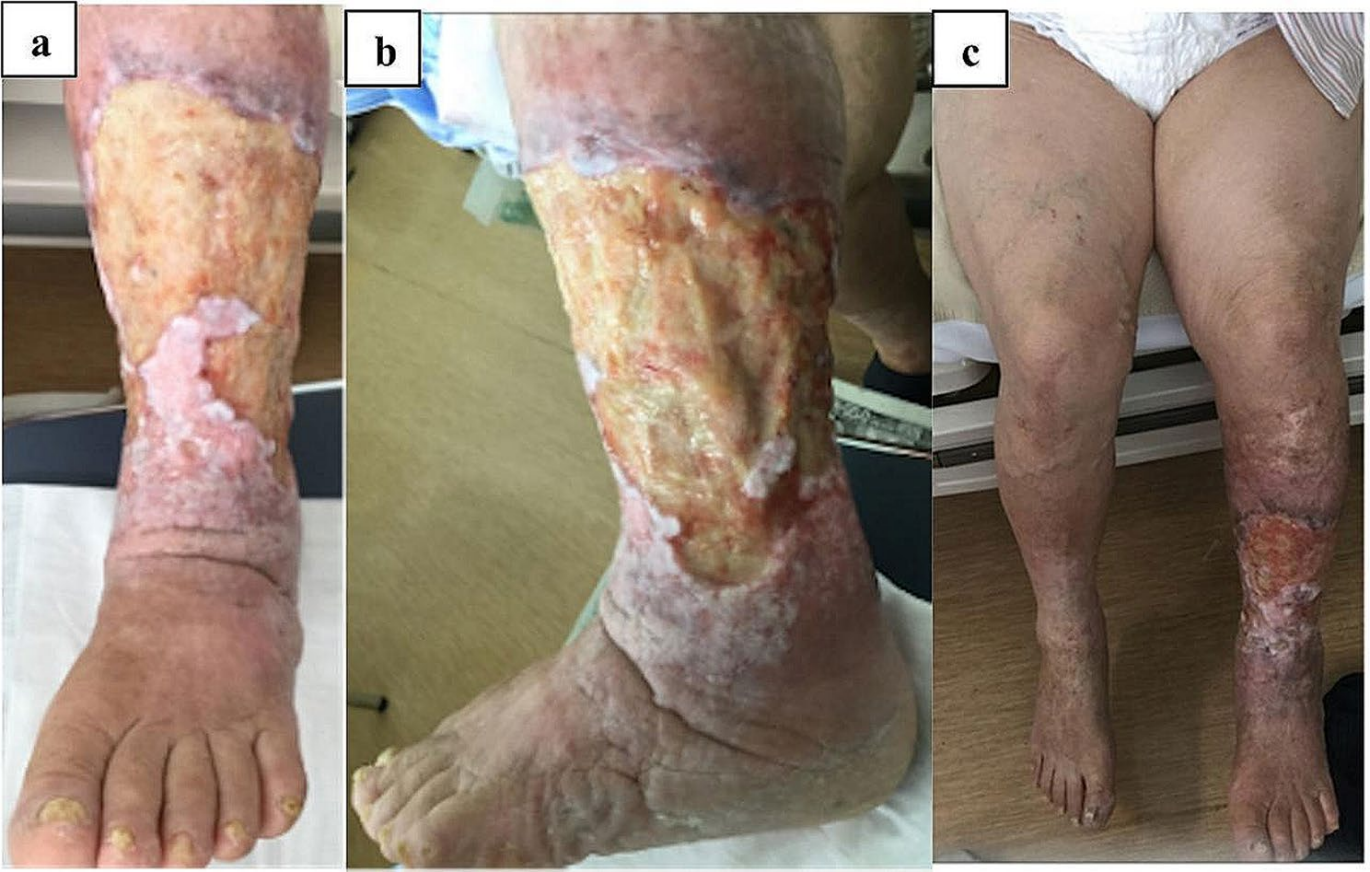
**a**, **b**, **c**. Intractable left leg ulcer and varices of bilateral legs

The patient was an 86-year-old woman with asymptomatic severe aortic stenosis (AS). She had scleroderma with intractable left leg ulcers, bilateral leg varices, hypertension, and diabetes mellitus. She had been followed up for 2 years by plastic surgeons. Due to delayed wound healing in the lower extremities, plastic surgery consulted cardiology, who diagnosed very severe aortic valve stenosis. On preoperative echocardiogram, aortic valve peak velocity was 5.5 m/s, and the mean pressure gradient was 76 mmHg; the aortic valve area was 0.54 cm^2^ in planimetry and 0. 86 cm^2^ in the continuity equation. Left ventricular internal end-diastolic and end-systolic diameters were 45 and 27 mm, respectively; ejection fraction was 71%. There was mild mitral and tricuspid valve regurgitation. Although her mean gradient was 76 mmHg, it is possible that the condition of the patient with scleroderma, leg ulcers, and chronic advanced venous insufficiency had an impact on the patient actual capacity and that she was asymptomatic. Despite being asymptomatic, surgery was planned because of very severe AS. Considering the possibility of the spread of infection from the lower extremity wound (Fig. 1), SAVR was performed via right anterior thoracotomy to avoid wound infection and mediastinitis. Cardiopulmonary bypass (CPB) was established with cannulation of the right femoral vein and artery (FA). Through the right third intercostal thoracotomy, left internal mammary artery and vein were ligated, and the fourth rib was dissected at the costochondral site. Aortotomy was attempted at 2 cm above the sinotubular junction. The aortic valve was tricuspid and showed calcific degeneration. Denaturation of noncoronary cusp (NCC) and right coronary cusp was particularly notable. The valve cusps were resected, the annulus calcification was removed, and the left ventricle was washed. A 19 mm bioprosthetic valve (INSPIRIS RESILIA Aortic Valve, Edwards Lifesciences, California, United States of America) was placed in the supraannular position. After confirming the patency of the coronary ostia, the aortotomy was closed. The heart was de-aired from the aortic vent, and the aorta was declamped.

**Fig. 2:**
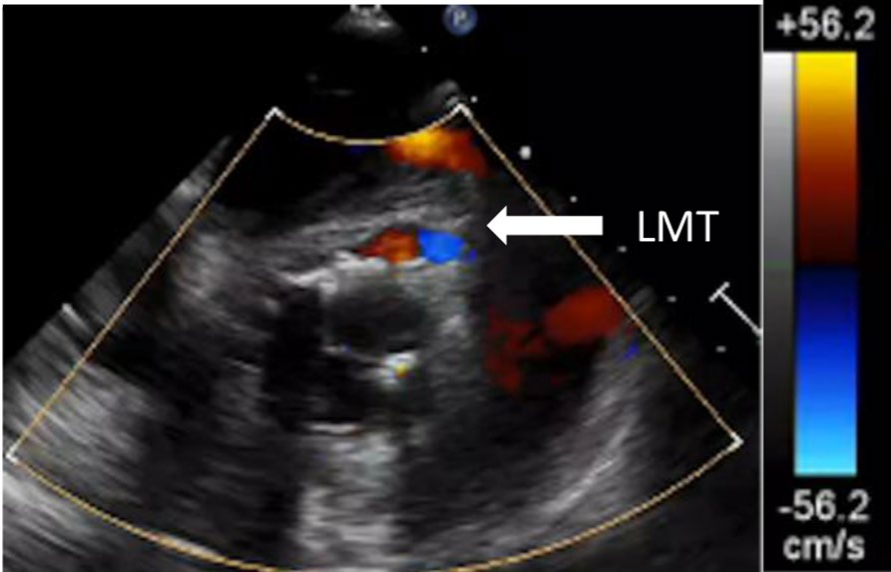
Transesophageal echocardiogram findings just after de-clump. More than 56.2 cm/s flow was detected in the left main trunk (LMT)

**Fig. 3:**
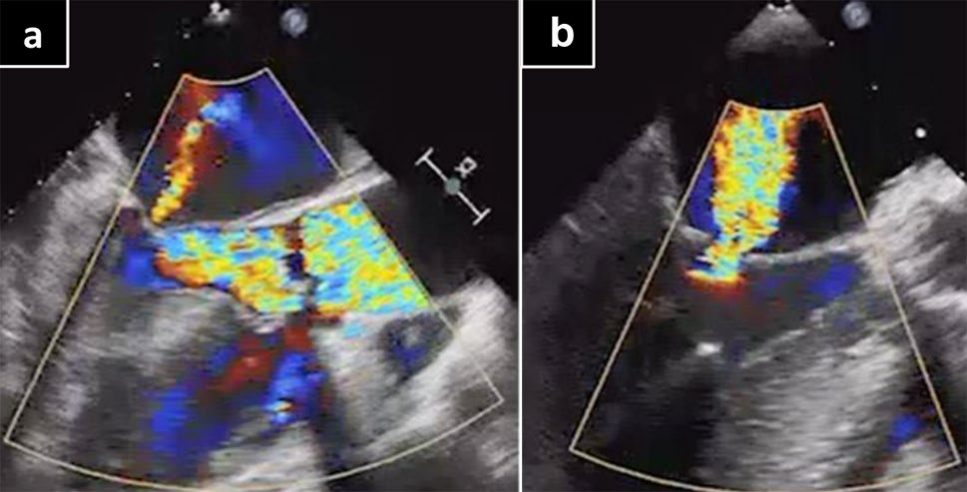
**a**, **b**. Mitral valve regargitation with central jet. (**a**) Preoperative and (**b**) intraoperative echocardiography

**Fig. 4:**
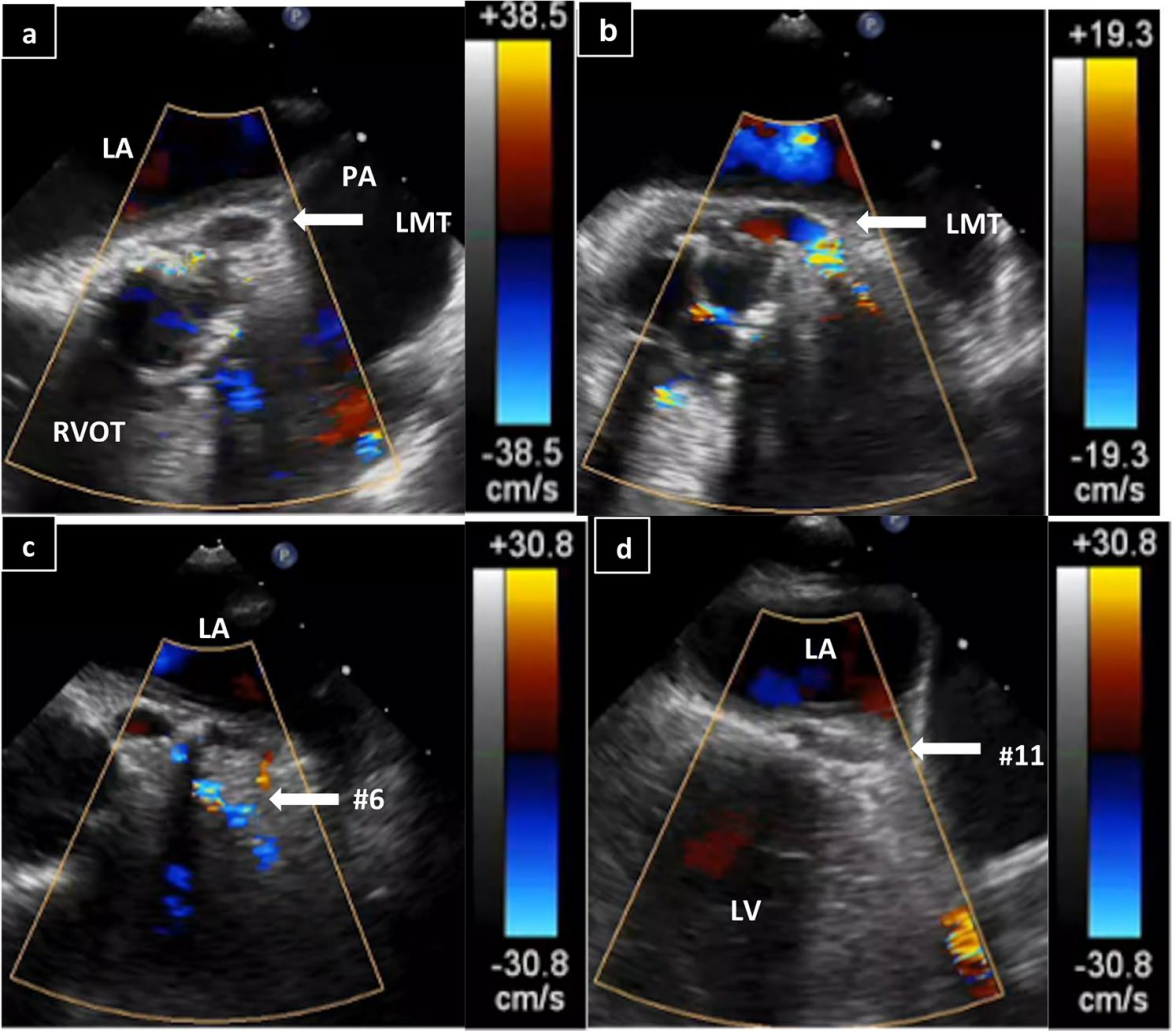
**a**, **b**, **c**, **d**. Transesophageal echocardiogram findings after asynergy. The left main trunk blood flow became undetectable in the range of 38.5 cm/s; 30-degree scanning angle. (**a**), but was picked up by reducing the blood flow range to 19.3 cm/s; 30-degree scanning angle. (**b**). Proximal left anterior descending artery (#6) flow was narrowly detectable; 30-degree scanning angle. (**c**), and no distal circumflex artery (#11) flow was observed; 120-degree scanning angle. (**d**)

The patient was placed off-pump uneventfully with dobutamine (2 µg/kg/min). TEE showed that the right and left coronary arteries were patent (Fig. 2). However, 20 min after being placed off-pump, her blood pressure decreased; TEE indicated poor contraction of the anterior and lateral wall and moderate mitral regurgitation (Fig. 3). Moreover, the coronary flow was decreased, and there was loss of coronary artery flow in left main trunk (LMT), #6, 11 (Fig. 4). TEE ruled out coronary or aortic dissection and postoperative ischemia caused by a malpositioned prosthetic valve occluding the coronary ostium. However, color Doppler showed mean flow velocity of over 20 cm/s in the left coronary artery. Based on focal dysfunction, coronary artery ischemia was suspected. We switched arterial cannulation from FA to ascending aorta, and the intra-aortic balloon pump (IABP) was inserted from FA. Under 1:1 IABP support, blood pressure and TEE showed improved contraction as well as flow velocity in the left coronary artery. Her vital signs stabilized after infusion of dobutamine (10 µg/kg/min) and adrenaline (1 µg/kg/min).

Stenosis caused by an embolus was suspected because the stenosis had moved slightly and no dissectional flap was observed. In addition, because high-intensity lesions such as calcification were not observed on ultrasound, a thrombus or fat embolism was suspected.

These findings suggested that catheter intervention was feasible. As the patient had scleroderma with an intractable ulcer on the left lower leg, we opted for PCI instead of bypass surgery, as transfer of the patient to the catheterization room for coronary angiography (CAG) was deemed to be safe.

**Fig. 5:**
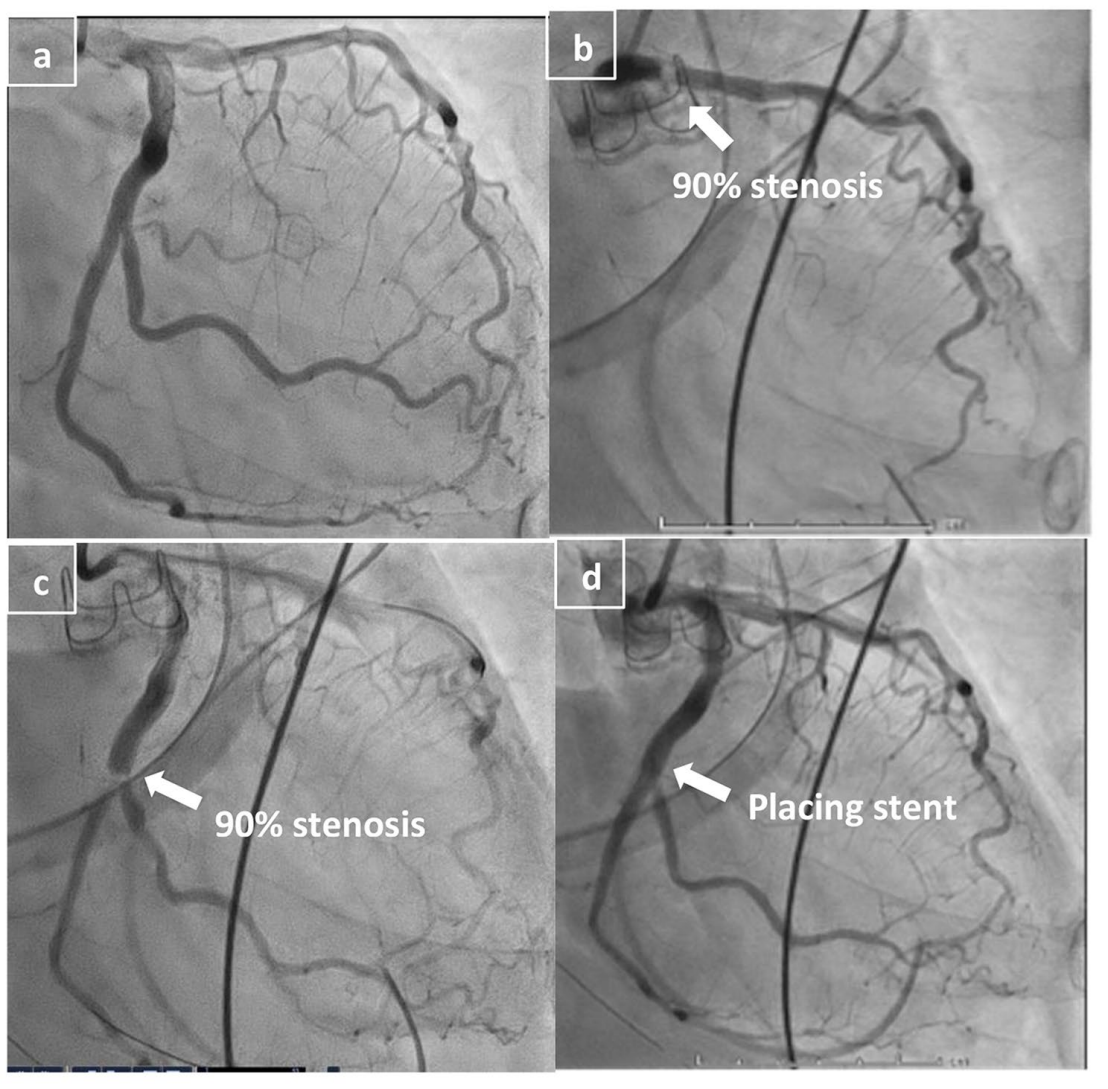
**a**, **b**, **c**, **d**. Preoperative coronary angiogram (**a**). Intraoperative coronary angiogram showing 90% stenosis in the proximal left anterior descending artery (**b**) and the nonenhanced circumflex artery (**c**). After successful passage of the wire and balloon expansion, the circumflex artery (LCx) was opened, and the stent was placed (**d**)

CAG showed obstruction at the entrance of the left circumflex artery, suggesting the presence of an embolus (Fig. 5.b). Intravascular ultrasound showed a low echoic embolus. Attempted aspiration with Thrombaster III (KANEKA MEDICAL, Tokyo, Japan) was unsuccessful. The embolic material was displaced by wire insertion to the distal lesion (#13) (Fig. 5.c), and a balloon expander (AsahiNC Kamui 2.5 × 12 mm, ASAHI INTECC, Aichi, Japan) was used to dilate the lesion to 20 atm for 30 s. However, no morphological change was observed in the stenotic lesion. A drug-eluting stent 3.5 × 15 mm (XienceSkypoint, Abbott, Chicago, Illinois) was placed at #13 (Fig. 5.d). On postoperative day (POD) 1, an echocardiogram showed trivial central aortic regurgitation, and left ventricular internal end-diastolic and end-systolic diameters were 43 and 32 mm, respectively; ejection fraction was reduced to 49% without asynergy. Her vitals were stable on POD2, and IABP was removed. Postoperative lymphorrhoea in the right inguinal region was treated with direct lymphostasis by suture ligation, and the patient was discharged on POD21. A 1-month follow-up echocardiogram indicated no decline in ejection fraction (48%) without asynergy and no signs of surgical site infection, and the condition of her leg ulcers improved slightly.

## Discussion

The reported incidence of coronary ostial stenosis after SAVR ranges between 0.1% and 5% [1, 2]. Ischemic symptoms typically present within 3–6 months after SAVR. Funada et al. [3] and Somopopolou et al. [4] suggested that the most likely pathophysiological mechanism of coronary artery stenosis in the late stage of SAVR is posttraumatic fibrous intimal proliferation caused by coronary ostia cannulation for direct cardioplegia during the operation. Other potential technical problems [1, 5] that lead to the obstruction of a given coronary osmium include inaccurate size, improper positioning of the prosthesis, and suture line. There are only a few case reports concerning coronary stenosis as an acute complication after SAVR due to calcium-like material [2, 6], thrombus [7], or unknown emboli [8].

In such cases, abnormal wall motion was detected using TEE, and coronary problems were suspected and diagnosed via coronary angiography.

TEE can provide pivotal information during cardiac valve replacement surgery, including prosthetic valve motion, pathological leaks, findings related to other valvular diseases, closely evaluate the new relationship between the bilateral coronary ostium and new prosthetic valve [7], and aortic event such as aortic dissection [9] as well as to cardiac wall mortion.

The uniqueness of this case is that repeated TEE confirmed the moving stenosis site, which revealed that the cause was an embolus and that it was not a calcified lesion based on the echo brightness. Furthermore, by adjusting the Doppler sensitivity, it was possible to detect a slight flow, diagnosing a partial occlusion rather than a complete occlusion implying that the wire may be able to pass through. This suggested the possibility of suctioning out the embolus with a catheter, or even placing a stent. Because ECG can facilitate fast diagnosis of coronary problems, we were able to find the cause and consider the best surgical approach for this patient through patient observation with TEE.

CABG and PCI are the main treatment modalities for coronary obstruction by debris. The advantage of CABG is that it can be completed in the operating room where CPB can be used, thus improving safety. However, it is more invasive due to the need for graft harvesting, and CABG is not always safe. It can result in perioperative infarction, a high operative mortality rate, or a poor long-term outcome [10] PCI is a minimally invasive procedure that allows direct aspiration of the embolic material [11] or stent placement, precluding the need for grafting. In fact, several reports have shown good early and late outcomes with PCI and stent implantation [4, 12].

However, PCI entails a risk of sudden hemodynamic collapse due to the need to transfer the patient to the hybrid room or catheter room; moreover, there is a possibility of PCI failure due to the failure of passage of wire or stent expansion.

The most common cause of this after valve surgery is a simple air embolism, which should resolve with time and adequate circulatory support. If not, coronary occlusion is the cause, and to diagnose it properly, diagnostic coronary angiography is the gold standard. In this diagnosis and management process, TEE not only detected and diagnosed coronary occlusion but also suggested the feasibility of wire passage and stent expansion based on Doppler flow and echo intensity, thus allowing the choice of percutaneous coronary intervention and providing more information for treatment strategies as a next step.

In fact, information from TEE was particularly helpful in this patient as obtaining a saphenous graft would have been challenging due to lower extremity ulcers and bilateral varices; moreover, CABG would have necessitated conversion from anterior lateral approach to median sternotomy. Considering the possibility that emboli may move to the proximal site because of the turbulent flow due to the retrograde flow resulting from CABG, we decided that it would be better to press against the wall with a stent. Thus, repeated and continuous TEE conferred a distinct advantage in helping us avoid CABG and the benefit of PCI. There are some limitations. First, TEE cannot characterize the nature of embolus. Second, sudden hemodynamic changes may occur during transfer to the catheterization room or hybrid operation room. In case of unstable vitals, patient may be transferred with extracorporeal membrane oxygenation [2]. Third, even though SAVR was selected because the annulus size was small (19 mm) and calcification was quite severe, transcatheter aortic valve intervention could have been an appropriate treatment option [13]. Fourth, we could not remove the embolus and leave a possibility of embolism to another location. Fifth, intermammary artery harvesting can be done through anterior thoracotomy [14]; however, we could not perform it technically. Sixth, the long-term outcome of PCI in this patient is yet to be determined. Although it is not possible to arrive at a conclusion based on the experience of just one case, I hope that by reporting this case, knowledge will be gathered at many facilities and that a new monitoring method will be developed.

## Data Availability

No new data were generated or analyzed in support of this research.
